# Editorial: “Design, Modeling and Manufacturing of Scaffolds to Control Cell-Biomaterial Interactions in Tissue Engineering”

**DOI:** 10.3389/fbioe.2022.868362

**Published:** 2022-02-23

**Authors:** Elisa Mele, Daniele Tartarini, Lorenzo Moroni

**Affiliations:** ^1^ Materials Department, Loughborough University, Loughborough, United Kingdom; ^2^ Department of Computer Science, University of Sheffield, Sheffield, United Kingdom; ^3^ INSIGNEO Institute for in Silico Medicine, University of Sheffield, Sheffield, United Kingdom; ^4^ Complex Tissue Regeneration Department, MERLN Institute for Technology-inspired Regenerative Medicine, Maastricht University, Maastricht, Netherlands

**Keywords:** scaffolds, mathematical models, tissue engineering, 3D printing, membranes

The chemical composition and physical properties of scaffolds for tissue engineering significantly impact tissue regeneration by producing biochemical, physical, and mechanical signals that regulate cell fate. Research has been conducted to control these properties using a variety of approaches that include manufacturing methods such as lithography, phase separation/inversion, electrospinning, and additive manufacturing. The design of scaffolds that stimulate biological responses after implantation requires knowledge of the cellular processes underlying, among others, mechano-transduction, adhesive interactions, synthesis of extracellular matrix and proteins. Mathematical and computational modeling can provide the necessary framework to study phenomena at the appropriate spatio-temporal scales, which are not always readily observable. The processes driving cell population growth and dynamics, cell-cell interactions, cell-material interactions, nutrients transport, angiogenesis are crucial in the identification of the governing principles and rely on modeling to advance theoretical and experimental understanding. A synergy between manufacturing and modelling is required to further progress the tissue engineering sector towards the generation of functional tissues and organs for clinical translation.

This Research Topic focuses on recent advances around scaffolds for tissue engineering, *in-vitro* platforms to mimic complex cellular interactions, and mathematical models for tissues and organs. This collection of papers is formed of one review article, two mini review articles, and five original research articles.

The role played by porosity and pore size of scaffolds on cell growth and tissue repair was investigated by Liu et al. using nano-hydroxyapatite/polyamide 66 (n-HA/PA66) composite scaffolds with three different types of porosity. They analyzed variability on adhesion, growth, proliferation, and osteogenic differentiation of bone marrow-derived mesenchymal stem cells (BMSCs) when seeded onto the scaffolds. Osteogenesis and bone ingrowth were evaluated *in vivo* by implanting the scaffolds into cranial defects in an animal model. The study concluded that, to effectively control BMSCs differentiation and achieve bone repair, relatively large pore size (∼370 µm) and high porosity (>78%) were needed.

Polymeric scaffolds with up to 99% porosity and high interconnectivity can be manufactured by emulsion templating, which has been investigated only recently as a fabrication method for tissue engineering. Dikici et al. comprehensively reviewed the basic principles of this technique and the key properties of emulsion templated scaffolds. The authors focused on the requirements for the preparation of High Internal Phase Emulsions (HIPEs) and Polymerised High Internal Phase Emulsion (PolyHIPE), such as the selection of the appropriate chemicals for both the continuous phase (polymer or pre-polymer) and the internal phase (solvent) of the emulsions, with considerations on stabilizers (surfactants), initiators, crosslinking agents, and experimental conditions (temperature and mixing efficiency). The application of PolyHIPE scaffolds with improved biomimetic behavior was then analyzed for hard and soft tissue regeneration, and for the controlled release of drugs and bioactive molecules. The challenges to commercialization and clinical use of PolyHIPE scaffolds were discussed with reference to *in-vivo* evaluations, long-term stability, sterilization routes, and shelf life.

Salt particles, like emulsions, can be used as template to manufacture polymeric scaffolds with high porosity. Teixeira et al. prepared porous RGD-alginate scaffolds by combining particle leaching (sodium chloride as porogen) and freeze drying. Fibroblasts and human outgrowth endothelial cells were seeded onto the porous scaffolds to create a vascular stromal environment, where porosity promoted cell colonization and infiltration within the scaffolds. The scaffolds were then treated with alginate solutions containing epithelial cells and *in-situ* crosslinking of the alginate enabled the formation of cell-laden hydrogels inside the pores of the scaffolds. This was considered the parenchymal environment. The developed hybrid system supported epithelial morphogenesis in organoids/tumoroids and could be used as an *in-vitro* model of vascularized breast tumor to screen drugs or test anticancer therapies.

The importance of *in-vitro* models for cancer is highlighted in the work of Marino et al. too, where optically transparent membranes were studied to mimic the blood-brain barrier (BBB). The membranes were made of cellulose acetate and produced using two different techniques to obtain a range of pore sizes: vapor-induced phase separation (VIPS) and electrospinning. Both types of membranes were seeded with a co-culture of human astrocytes from healthy brain and human cerebral microvascular endothelial hCMEC/D3 cells that well adhered on the membrane surfaces and formed a biological barrier. The VIPS membranes were characterized by a homogenous distribution of nanopores, and hence hold potential as model to analyze how molecules or nanomaterials can cross the BBB; while the electrospun mats, which had micropores and were infiltrated by cells, could be used as model for the blood-brain-tumor barrier and invasion assays.

Electrospinning is a widely accepted approach to synthetically produce polymeric nanofibers and replicate fibrillar structures that are available in nature. Azimi et al. reviewed the main properties of cellulose fibers synthesized by bacteria, known as bacterial cellulose (BC), and their relevance in the field of otology, particularly for the treatment of tympanic membrane (TM) perforations. They discussed studies, including animal studies and randomized controlled trials, that show how BC membranes can be effective in regenerating small TM perforations. Furthermore, they summarize computational approaches to model fibrous BC networks and perforations of the TM.

The combination of fibrous materials and three-dimensional (3D) structures produced by additive manufacturing (AM) has led to novel 3D scaffolds with multi-scale hierarchical porosity. Smith et al. reviewed the recent literature on multi-layered architectures formed *via* layer-by-layer deposition of electrospun nanofibers and 3D printed elements, and on hybrid scaffolds printed from bio-inks reinforced with nanofibers. They discussed how cellular behavior, such as attachment, migration, and differentiation, was influenced by the co-existence of micro- and nano-features, and how the technologies analyzed could be applied to develop sophisticated tissue models.

A similar hybrid manufacturing approach was reported by Higuera et al. by combining AM with polymeric microparticles. They researched into polymeric pastes made of poly(ethylene oxide therephthalate)/poly(butylene terephthalate) (PEOT/PBT) microspheres and biocompatible binders to achieve control over the surface roughness of 3D printed scaffolds. They optimized the properties of the scaffolds by acting on the microspheres size, the type of binder, the concentration of microparticles relative to the polymeric carrier, and the sintering temperature. It was observed that by increasing the roughness of the scaffolds, proliferation and attachment of human mesenchymal stromal cells were promoted together with an increased metabolic activity.

The papers of this Research Topic also discuss how the porosity of scaffolds modulates perfusion of nutrients and infiltration of cells. Control over mass transport, nutrient delivery and waste product removal is relevant also to organoid production, as described by Ellis et al. The authors developed a mathematical model that provided insight into the mass transport mechanisms regulating the proliferation rate of organoids in bioreactors. The mathematical model considered parameters such as inlet flow rate, cell seeding density, transport and spatial distribution of key metabolites (glucose and lactate), to inform the design of bioreactors for the reproducible and scalable generation of organoids.

In summary, this collection of articles covers recent advances on manufacturing scaffolds for tissue engineering and modelling cellular behavior. Emerging technologies have been analyzed and future directions to further progress in these areas are highlighted. It is expected that this Research Topic will inspire new research activities to further advance the design, modelling and manufacturing of scaffolds to control cell-biomaterial interactions in tissue engineering.

